# Nutritional and Non-Nutritional Strategies in Bodybuilding: Impact on Kidney Function

**DOI:** 10.3390/ijerph19074288

**Published:** 2022-04-03

**Authors:** Victoria Tidmas, Jon Brazier, Janine Hawkins, Scott C. Forbes, Lindsay Bottoms, Ken Farrington

**Affiliations:** 1 Centre for Research in Psychology and Sports Science, De Havilland Campus, University of Hertfordshire, Hatfield AL10 9EU, UK; v.tidmas@herts.ac.uk (V.T.); l.bottoms@herts.ac.uk (L.B.); 2 Centre for Health Services and Clinical Research, De Havilland Campus, University of Hertfordshire, Hatfield AL10 9EU, UK; j.hawkins3@herts.ac.uk (J.H.); ken.farrington@nhs.net (K.F.); 3 Department of Physical Education Studies, Brandon University, Brandon, MB R7A 6A9, Canada; forbess@brandonu.ca; 4 Renal Unit, Lister Hospital, East and North Herts Trust, Stevenage SG1 4AB, UK

**Keywords:** bodybuilding, kidney function, nutritional supplements, androgenic steroids, hypervitaminosis

## Abstract

Bodybuilders routinely engage in many dietary and other practices purported to be harmful to kidney health. The development of acute kidney injury, focal segmental glomerular sclerosis (FSGS) and nephrocalcinosis may be particular risks. There is little evidence that high-protein diets and moderate creatine supplementation pose risks to individuals with normal kidney function though long-term high protein intake in those with underlying impairment of kidney function is inadvisable. The links between anabolic androgenic steroid use and FSGS are stronger, and there are undoubted dangers of nephrocalcinosis in those taking high doses of vitamins A, D and E. Dehydrating practices, including diuretic misuse, and NSAID use also carry potential risks. It is difficult to predict the effects of multiple practices carried out in concert. Investigations into subclinical kidney damage associated with these practices have rarely been undertaken. Future research is warranted to identify the clinical and subclinical harm associated with individual practices and combinations to enable appropriate and timely advice.

## 1. Introduction

Bodybuilding, or “the use of progressive resistance exercise to develop muscle building by hypertrophy”, has become increasingly popular both recreationally and competitively [1]. Unlike other sports, bodybuilding success is judged on appearances of muscular definition and symmetry rather than athletic ability [2]. Resistance training, including non-competitive-style training, is recommended for both athletes and the general population to improve physical performance, appearance, strength, rehabilitation, health, and aid in weight management [3,4]. Competitive bodybuilding pushes the limits of resistance training adaptations and requires dedication to rigorous training and strict dietary regimes [1,5]. Over 95% of bodybuilders use dietary supplements [6], with the two most common being creatine monohydrate and protein [5,6]. Non-nutritional, performance-enhancing drugs, such as veterinary-grade vitamin supplementation, often in alarming quantities, and/or anabolic–androgenic steroids (AAS) are also used [6,7,8]. Most of the extreme nutritional, drug and training strategies used in bodybuilding are derived from non-evidence-based sources [1,2,5], giving rise to increasing concerns about their potential adverse health impact [9]. Adverse renal effects include increased risks of acute kidney injury (AKI) and a number of conditions that may lead to chronic kidney disease (CKD) [8,10,11]. In this review, we will explore links between dietary and non-nutritional supplementation strategies observed in bodybuilders and their impact on kidney function, injury and disease.

## 2. Materials and Methods

**Figure 1:**
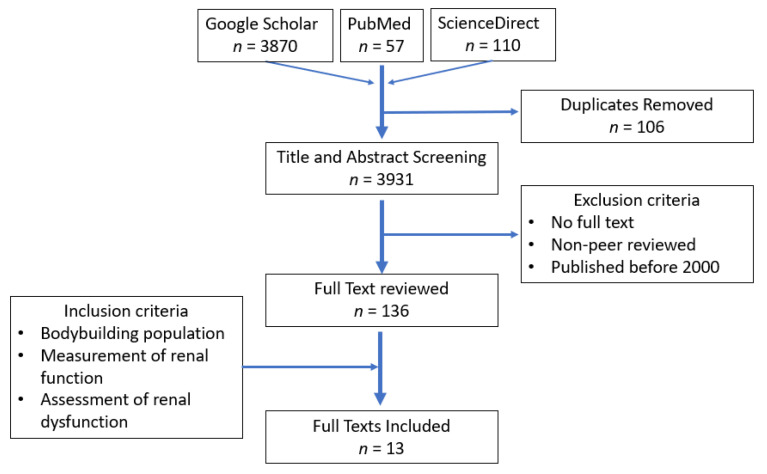
Flow diagram describing the search methodology used to obtain the research studies for this review.

Google Scholar, PubMed and ScienceDirect were accessed to conduct an online literature search. Although this review is narrative in design, the search strategy and parameters closely followed the PRISMA statement guidelines [12], to promote and focus the findings to a high quality, ensuring scientific and accurate discussion of areas of importance. As such, the key search terms and phrases used were formed from a combination of the following: ‘bodybuilding’, ‘nutrition’, ‘protein’, ‘creatine’, ‘supplements’, ‘anabolic-androgenic steroids’, ‘kidney function’, ‘acute kidney injury’ and ‘chronic kidney injury’. The search method is outlined in Figure 1, where firstly duplicates were removed, and then the title and abstracts were screened for relevance and significance. Additionally, papers were excluded if access to full texts was denied, published by a non-peer-reviewed journal, and if published prior to 2000, to ensure that only the most relevant and up-to-date literature and results are included. To further ensure that the papers reviewed were significant, the compiled full texts were only included if the inclusion criteria were met, namely, concerning a bodybuilding population, measurements of renal function and subsequent assessment of renal dysfunction or injury if applicable. The search resulted in 13 significant full-text articles discussed throughout this review. The reference lists of these key articles were scanned, and additional articles of interest not found by the electronic database search were included or discussed throughout the review.

## 3. Results

Thirteen studies published since 2000 described kidney disease in 75 bodybuilders (Table 1; the full details of the renal conditions and dietary and supplementary practices can be seen in Supplementary Tables S1 and S2). Most studies (eight) were case reports and the remainder case series. A range of kidney conditions were involved. There were 25 cases of acute kidney injury (AKI)/acute tubular necrosis (ATN), 20 had focal segmental glomerular sclerosis (FSGS) and 10 nephrocalcinosis. Other conditions reported were acute interstitial nephritis (AIN) (5 cases), nephrosclerosis (5 cases), chronic interstitial nephritis (3 cases) and an assortment of other glomerulonephritides (7 cases). Each condition was usually associated with multiple bodybuilding practices, nutritional and non-nutritional, which will be outlined in the following sections.

## 4. Discussion

### 4.1. Measurement of Kidney Function

**Figure 2:**
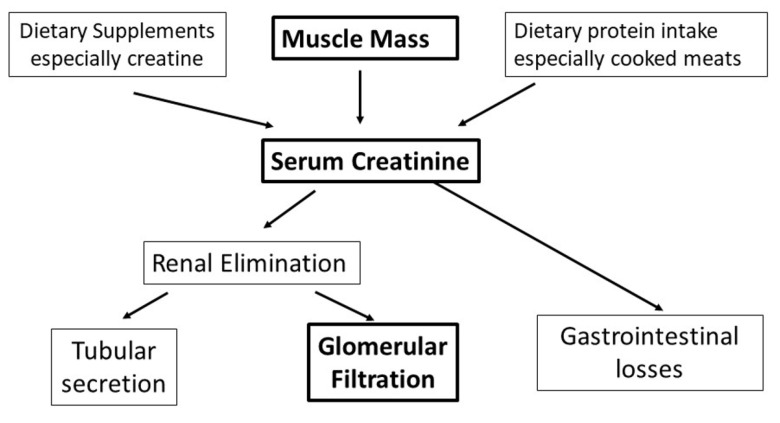
Determinants of serum creatinine.

Estimation of kidney function in clinical practice is based largely on measurements of serum creatinine. Creatinine is the end-product of creatine and creatine phosphate metabolism. Creatine is generated predominantly in the kidney and liver from glycine, arginine and methionine, and transported to other tissues, particularly skeletal and heart muscles, where it undergoes phosphorylation by creatine kinase to creatine phosphate [13]. Creatine phosphate provides a readily available source of energy, particularly during the early phases of intense muscular contraction. Creatinine is produced by non-enzymatic anhydration of creatine within muscle [14]. Skeletal muscle has the highest concentration of creatine and creatine phosphate (~95%) and is thus the primary source of creatinine generation [14,15], although high-protein diets—particularly those containing cooked meats—are another source of creatinine. The kidney is the main route for creatinine elimination, though there may also be low-level gastrointestinal removal. Creatinine is freely filtered by the glomerulus but also undergoes tubular secretion, the rates of which can be unpredictable and influenced by many drugs, including cimetidine and trimethoprim [14,15]. Figure 2 illustrates the main determinants of serum creatinine.

Hence serum creatinine values, though key measures of kidney function, must be interpreted with reference to muscle mass. Measurement of creatinine clearance is a more reliable indicator of the glomerular filtration rate (GFR), though it overestimates the gold standard inulin clearance by around 10% [14]. It also involves lengthy, timed urine collections, the impracticability of which in routine practice has favoured the use of estimates of GFR (eGFR) based on empirical modifications of serum creatinine. The simplest equation also involves functions of age, sex and ethnicity to help correct for creatinine generation [16].

Utilising cystatin C could prove to be a more reliable and accurate measure of kidney function. It has fewer non-GFR determinants, and, in particular, is related to neither muscle mass nor diet, as is serum creatinine [17]. However, it has been reported to be increased in the presence of anabolic androgen steroid (AAA) use [18]. Hence, both serum creatinine and cystatin C present issues in the quest for accurate assessment of kidney function in the bodybuilding population. It has been recommended, though, that for individuals with extremes of muscle mass or diet, cystatin C should be measured and eGFR reported as eGFRcr-cys, or that GFR should be measured directly using a clearance procedure [19]. Likewise, for the assessment of albuminuria in patients with extremes of muscle mass or diet, measurement of the albumin:creatinine ratio should be supplemented by the albumin excretion rate measured from a timed urine collection [19]. Few studies of kidney health in bodybuilders have met these criteria. Other promising biomarkers of kidney health may allow detection of early damage (such as plasma NGAL and urinary KIM-1 for risk of developing AKI), localisation of injury and prediction of disease progression [20]. Their use in bodybuilding studies is rare.

### 4.2. Protein Supplementation

UK government guidelines recommend protein intakes of 0.75–0.8 g/kg/day [21]. This is increased up to 1.4–2.0 g/kg/day for athletes [22]. This is safe, aids training adaptations and optimises building and maintenance of muscle mass [22]. Protein is essential for muscle anabolism. Higher intakes can be beneficial for strength and power athletes, including bodybuilders [23]. Use of high-protein diets is widespread in bodybuilders presenting with kidney dysfunction [7,24,25,26,27,28] (Table 1). Hartung reported a protein intake of 2 g/kg/day in a bodybuilder presenting with end-stage kidney disease due to FSGS [27]. Herlitz found that 8 of 10 bodybuilders consumed high-protein diets (2.8–5.1 g/kg/day) [28], and nine had FSGS. Almukhtar found that four bodybuilders with AKI had a total protein intake of 3.2–4.2 g/kg/day [25]. The protein intake of 22 bodybuilders with a range of kidney conditions (Table 1) was reported as an incredible 20–30 g/kg/day [26]. All these intakes are equal to or greater than the highest intake recommended for sports participation. In none of these studies was high protein intake thought to be the dominant contributor to kidney dysfunction. In most cases multiple other behaviours were also implicated.

Other studies have found no detrimental effects of isolated high-protein diets, albeit consumed for relatively brief periods [29,30]. In five healthy resistance-trained men, taking 2.5 ± 1.0 to 3.5 ± 1.4 g/kg/day for 2 years, there was no significant effect on kidney function [29]. Similarly, in a randomised crossover study in 14 resistance-trained men consuming 2.51–3.32 g/kg/day for one year, no harmful renal effects were observed [30]. In both studies the only parameters reported relating to kidney function were serum creatinine, blood urea nitrogen and eGFR.

There is evidence that high-protein diets can cause issues for those with pre-existing kidney insufficiency [31]. Dietary protein is a key physiological modulator of renal function. High-protein diets can cause glomerular hyperfiltration and increase the intraglomerular pressure, potentially leading to structural damage [32]. Acute haemodynamic effects of high protein intake can be seen in the short term in healthy individuals [33]. In longer-term epidemiological studies, higher levels of protein intake are associated with a more rapid decline in kidney function [34]. A strong case has been made for lower protein intakes in patients with CKD [35].

Bodybuilders generally employ different nutritional strategies [36] during specific phases of training, such as bulking (calorie excess to increase muscle mass) or cutting (pre-competition calorie reduction to reduce body fat and increase muscle definition) [1,37]. Although carbohydrates are the main macronutrient manipulated in the week(s) prior to competition, high protein consumption is maintained [36]. Bodybuilders observed by Gentil [37] increased their daily protein intake from 2.5 g/kg/day during bulking to ~3.0 g/kg/day when cutting. In contrast, Maestu found that baseline protein intake was slightly reduced from 2.68 g/kg/day to 2.48 g/kg/day 3 days prior to competition [38]. Similarly, Lenzi found that 4.16 ± 1.28 g/kg/day of protein was ingested when bulking, reducing to 3.56 ± 1.43 g/kg/day during cutting [1]. None of the studies detailing the protein intake of bodybuilders in relation to kidney dysfunction [7,24,25,26,27,28] stated the training phase of the individuals involved. Multiple assessments of intake during different training phases will be necessary to establish the precise levels of intake and define any risks involved.

Overall, there is little direct evidence that high-protein diets contribute to an increased risk of kidney dysfunction in healthy bodybuilders. In the cases of FSGS reported, concomitant use of other potentially culprit practices were the norm. Risks are likely higher in those with pre-existing kidney impairment. However, the evidence base is poor, and further research necessary.

### 4.3. Creatine Supplementation

Dietary sources of creatine include fish, meat and dairy products, which contribute to an intake of 1–2 g/day. Creatine can also be synthesized endogenously in the kidney and liver [13]. Creatine supplementation has become increasingly popular in athletes of all levels, due to its perceived performance-enhancing properties, such as increasing lean muscle mass and strength, improved post-exercise recovery and injury prevention [39]. It is one of the most commonly used non-hormonal supplements in bodybuilders [40]. Typically, four equal doses (4 × 5 g) are ingested daily (~0.3 g/kg/day or 20 g/day) for 5–7 days to load muscles with creatine. Following this, 3–5 g/day is used as maintenance. This dosing strategy has been shown to augment resistance-training gains [39]. Seven studies have identified creatine use in bodybuilders with renal dysfunction, although only five outlined specific dosing strategies (Table 1). Maintenance doses consumed were variable with respect to those recommended [25,41,42,43]. In an extreme case it was reported that the individual consumed 210 g/day, which is 22 times greater than the recommended maintenance dose [26]. Again, creatine supplementation was rarely used in isolation. Only one study [41] specifically stated denial of use of AAS, NSAIDS and diuretics, while two studies did not mention any other bodybuilding practices, such as nutritional intake or use of other agents [42,43]. The kidney conditions most frequently associated with creatine supplementation in bodybuilders are acute interstitial nephritis (AIN) and acute tubular necrosis (ATN), which have occurred in individuals consuming relatively low doses [42,43]. It is possible that other agents were also being consumed—though none were reported—or that excipients in the creatine preparations were the real culprits. In keeping with these possibilities, prolonged use of high maintenance doses of 9.7 ± 5.7 g/day for up to four years has been shown to have no apparent detrimental effects [44]. Similarly, creatine supplementation together with a high protein intake (1.2–3.1 g/kg/day) and resistance training had no apparent effect on creatinine clearance and albuminuria [45].

Within tissues or solutions, creatine is gradually and irreversibly converted to creatinine, which is filtered by the kidneys and excreted via urine [46]. However, creatine can also be converted into sarcosine, resulting in the production of methylamine, and via subsequent deamination, the formation of formaldehyde, which is potentially cytotoxic [47]. Creatine supplementation (21 g/day for 14 days) significantly increased the serum levels of methylamine [46] and formaldehyde [48], although both remained within the upper limits of normal [49]. Furthermore, Candow and colleagues examined the effect of a relative low dose of creatine (0.1 g/kg) taken on resistance-training days (3 times/week for 10 weeks) versus the placebo, in older healthy men [50]. There were no significant changes over time, and no group differences, in urinary formaldehyde excretion.

Creatine homeostasis is dependent on dietary creatine intake in conjunction with endogenous creatine production to offset creatine degradation (1.6–1.7% of the total creatine pool is degraded daily). The capacity of renal arginine:glycine amidinotransferase (AGAT), a key enzyme involved in creatine synthesis, is progressively decreased with progression of CKD and is virtually absent in dialysis patients [51]. Furthermore, plant-based foods are increasingly recommended for individuals with kidney dysfunction since they contain smaller amounts of saturated fatty acids, protein and absorbable phosphorus. They also generate less acid and are richer in fibre than meat. It has been suggested that loss of endogenous synthesis capabilities and reduced consumption of creatine rich foods can result in creatine deficiency. Creatine deficiency is associated with clinical manifestations similar to sarcopenia, with fatigue, impaired quality of life and impaired cognition [51]. Future research should examine the impact of creatine-rich foods or creatine supplementation on individuals with CKD. Overall, evidence that creatine supplementation increases the risk of developing kidney damage in bodybuilders is inconclusive. Further research is necessary to ascertain whether creatine supplementation is a true cause for concern.

### 4.4. Anabolic Androgenic Steroids

Anabolic androgenic steroids (AAS) are used by professional athletes such as bodybuilders and powerlifters to enhance performance, increase muscle mass and decrease body fat [9]. Their use has become increasingly common in bodybuilding and amongst non-competitive athletes seeking an increased muscular appearance [10,26,52]. Illicit use by recreational sportspeople has overtaken that for athletic use (overall prevalence reported as, respectively, 18.4% and 13.4%) [53]. However, ascertaining the true prevalence is challenging since these agents are often taken secretly and research using anonymised questionnaires can only provide a very approximate insight [9,28,52]. There has been an alarming increase in AAS popularity worldwide. The global lifetime prevalence in 2014 was reported as 3.3%, though there were wide regional variations, the highest being in the Middle east (21.7%) followed by South America, (4.8%) and Europe (3.8%) [53]. Use of AAS has been deemed to pose a significant public health issue [9,54]. All major sports organisations have banned their use [9,26].

A recent study assessed the use of performance-enhancing drugs, mainly AAS, in bodybuilders. Current users had a higher prevalence of AKI than previous users and non-users, suggesting that individuals using these agents may be at an increased risk of kidney failure [10]. However, though use of AAS has been associated with a range of kidney conditions, including AKI, AIN, FSGS and nephrosclerosis, in all cases other bodybuilding practices were also being followed (Table). Of the 13 articles relating to bodybuilding (Table 1), three did not specify whether AAS were used [42,43,55] and in only two was their use denied [41,56]. The remaining eight confirmed a positive history of AAS use, although only four detailed specific quantities, type and duration of usage (Table 1). The training phase may also affect the AAS strategy employed [37].

An Endocrine Society scientific statement has warned that the use of AAS as performance-enhancing agents can lead to the development of significant complications, including FSGS, though cardiovascular risks outweigh those to the kidney [40,52]. FSGS may require a longer exposure to manifest [26], though subclinical kidney injury may occur earlier [18].

AAS have the potential to affect multiple pathways linked to kidney injury though evidence is largely limited to data from animal studies [40,57]. Androgens stimulate the renin–angiotensin–aldosterone system (RAAS), resulting in increased blood pressure and increased water retention via tubular reabsorption of salt and water [58]. Increased production of endothelin can result in both afferent and efferent vasoconstriction and increased mitogenic activity [57,59]. Increased RAAS activity and endothelin can also increase oxidative stress via NADPH oxidase [57]. Androgens can also promote apoptosis within the renal tubular cells by activating the caspase-dependent apoptotic pathway, contributing to the development of kidney fibrosis [60]. Testosterone has also been implicated in the production of pro-inflammatory cytokines, such as interleukin-1b, tumour necrosis factor α and interleukin-6, which can accelerate the progression of CKD [61,62].

**Figure 3:**
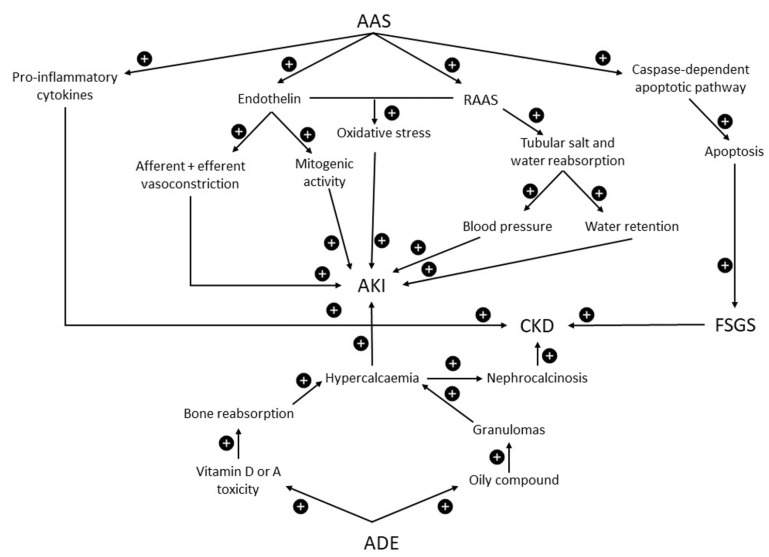
Possible mechanisms and pathophysiology of kidney injury due to exposure to AAS and intramuscular vitamins A, D and E. ADE = intramuscular vitamins A, D and E. AAS = anabolic androgenic steroids. RAAS = renin–angiotensin–aldosterone system. FSGS = focal segmental glomerulosclerosis. CKD = chronic kidney disease. AKI = acute kidney injury. + = increased effect.

The threat posed by AAS use to kidney health, as outlined in Figure 3, is better substantiated than protein and creatine supplementation, though again complicated by co-existent potentially harmful practices.

### 4.5. Vitamins

Vitamins are essential micronutrients with variable quantities required throughout a lifetime to support numerous physiological functions necessary for maintaining health. Vitamins, such as A and D, in large quantities can accumulate within the body, potentially resulting in hypervitaminosis [63]. Bodybuilders are known to inject high doses of vitamins A, D and E contained within an oily substance known as compound ‘ADE’, the consumption of which is damaging to human health and kidney function [54,64]. The first reported use of compound ‘ADE’ in humans was in Brazil in the late 1980s, though this practice may well have been in use sometime earlier [64]. Compound ‘ADE’ was originally used by veterinary professionals to treat cattle and horses experiencing vitamin deficiency and infections, in quantities not exceeding 5 mL per 120-day fattening period for cattle. Bodybuilders often exceed this [11]. The main reason that compound ‘ADE’ is used by bodybuilders is not for the vitamins per se but the oily carrier, which when injected into muscle produces a foreign body reaction and a local false hypertrophy [55], increasing the volume of the muscle through local fluid retention rather than muscle anabolism [64].

Of the six studies that report kidney conditions in relation to vitamin usage, four specifically outline the quantity, dosage and duration of use (Table 1). The quantities of ‘ADE’ that the bodybuilder used were far greater than those recommended for cattle. The resulting kidney pathologies ranged from the more common hypercalcaemia and secondary AKI, which after discontinuation of vitamin supplementation resulted in near complete renal recovery [7,56,64,65], to the development of nephrocalcinosis and CKD [7,55,64]. Abundant deposits of calcium phosphate can occur within the tubulointerstitium, often associated with irreversible interstitial fibrosis and tubular atrophy [11,64].

The development of vitamin D toxicity resulting in hypercalcaemia necessitates significantly increased intakes of 20,000–30,000 IU per day [66,67]. The bodybuilder’s vitamin D intake reported by Rocha (Table 1) was higher, at ~40,000 IU/day, resulting in a 25(OH)D level of 150 ng/mL, somewhat less than previously reported levels associated with vitamin D toxicity of ≥200 mng/mL [66,67]. Hence, it was proposed that vitamin A intoxication was the factor posing the greater influence on the hypercalcaemia and AKI presented by this particular patient [56]. Vitamin A intoxication is rare but known to cause hypercalcaemia by increasing osteoclast-mediated bone resorption [68,69]. However, in the case described by Ronsoni (suS2), the vitamin A levels were elevated but still within the upper limit of normal [65]. So, the vitamins within compound ‘ADE’ may not be the only factors responsible for hypercalcaemia. The use of cosmetic injections consisting of silicone, paraffin and polymethyl methacrylate have been associated with the development of hypercalcaemia [11,55,70]. These agents support the formation of granulomas that increase the 1-α-hydroxylase activity, thus promoting formation of 1,25-OH-D. The reduction in calcium levels in response to glucocorticoids provides some evidence to support this mechanism [68]. Nevertheless, there is strong evidence that use of compound ADE can cause hypercalcaemia, AKI and nephrocalcinosis, as displayed in Figure 3.

### 4.6. Non-Steroidal Anti-Inflammatory Drugs (NSAID)

NSAIDs are easily accessible, and their use is highly prevalent, accounting for around 5% of prescribed treatments worldwide [71]. NSAIDS are popular in sports medicine [71,72], with a self-reported prevalence of up to 50% among athletes [73]. They can counteract muscle inflammation, pain and soreness during, and in response to, exercise [74,75], and thus improve performance by increasing pain tolerances and delaying fatigue [75,76]. However, using high-dose NSAIDs has been shown to compromise training adaptations, reducing increments in muscle strength and hypertrophic gains from resistance exercise [72,77], thus potentially reducing performance. Regular high-dose NSAID administration significantly increases risks, in particular predisposing to the development of AKI and accelerating the progression of CKD [78]. In only three studies [28,41,56] (Table S2) was the use of NSAID specifically denied. The remaining studies did not report this practice (Table 1). Use of NSAIDS is likely to be an underappreciated factor in reports of kidney damage in bodybuilders.

### 4.7. Dehydration and Diuretics

The final days before bodybuilding competitions has been coined ‘peak week’ [36], during which the majority of competitors were found to implement strategies to maximise their physical appearance for aesthetic purposes [79,80]. These strategies often include altered exercise regimes; macronutrient, water and electrolyte intakes to enhance muscle glycogen content; minimising subcutaneous water; minimising abdominal bloating; and optimising muscle definition [36]. While natural methods can be used to achieve these objectives, bodybuilders often self-prescribe potentially harmful dosages of drugs, such as diuretics and insulin [36], which have resulted in dangerous outcomes, such as hypokalaemic paralysis [81]. Many bodybuilders acknowledge that these strategies pose increased health risks, but the measures are perceived as necessary and temporary [36]. The majority of strategies involve dehydration and sodium depletion in the final days before competition [79,80,82], which have been linked to increased risk of kidney dysfunction [83,84]. Only three of the studies (Table S2) specifically established a negative history of diuretic use [28,41,56]. Such practices may well pose a risk to kidney function, particularly in hot climates [25].

### 4.8. Other Supplements/Stimulants and Exertional Rhabdomyolysis

There are many other dietary supplements (DS) easily available to the general population [85] that were not found/highlighted within the scope of the literature search of this review, although they should not be dismissed. One DS of interest used by bodybuilders is “Hydroxycut”, a weight-loss and muscle-building product, comprising of Garcinia cambogia, Cissus quadrangularis, caffeine, Ma Huang (ephedra) and green tea in various quantities over the years of its production [86]. Caffeine is also considered the most popular pre-workout stimulant for bodybuilders, whereby improvements in strength performance requires large supplementation of ~5–6 mg/kg [2,87,88] at the upper range of the safe caffeine dosage of ~6 mg/kg [89]. Chappell et al. (2018) found that bodybuilders consume large amounts of energy and hot drinks, resulting in several bodybuilders exceeding the recommended safe dosage of caffeine [90]. Both consuming “Hydroxycut” [91,92] and caffeine [93,94,95] have been linked to the development of rhabdomyolysis. Therefore, bodybuilders practicing strenuous exercise while also taking these supplements/stimulants could be at increased risk of exertional rhabdomyolysis [96,97]. This is significant, as exertional rhabdomyolysis may also lead to the development of acute renal failure and multiple other organ dysfunctions [98,99]. Exertional rhabdomyolysis is a pathophysiological condition where musculoskeletal cell damage occurs due to intense or excessive exercise indicated by increased creatine kinase or myoglobin levels entering the blood via damaged cell membranes [100]. Myoglobin is said to be the main culprit of renal injury due to rhabdomyolysis, as it is excreted by the kidneys causing urine to be stained red or brown [101]. Although the exact mechanism of the myoglobins’ role in renal dysfunction and subsequent injury is uncertain, some evidence suggests that vasoconstriction of intrarenal blood vessels, direct oxidant and ischemic tubule injury and obstruction of tubular tissues may all be impacted, leading to acidic urine, renal obstruction and eventually acute renal failure if left untreated [101,102]. The three main signs and symptoms of exertional rhabdomyolysis are muscle aches, weakness and dark urine, and can often be overlooked as exercise-induced delayed-onset muscle soreness (DOMS) [99,102]. Bodybuilders should be informed of the risk of rhabdomyolysis and its repercussions on renal function if left untreated.

### 4.9. Practical Applications

The findings of this review bring certain bodybuilding practices to attention that should be monitored by athletes and coaches, as well as identified by medical professionals. Athletes with knowledge should make coaches aware of pre-existing renal deficiencies to advise their recommendations of nutritional and non-nutritional strategies. Increasing the occurrence of baseline and regular testing could provide more accurate measures of initial kidney function of athletes and can be used to identify previous unknown underlying conditions or significant changes during differing training phases, which could increase the risk of renal decline. The common and consistent concurrent use of multiple practises outlined in this review, including banned substances such as AAS, and the potentially underappreciated negative effect on renal function highlight the need to increase the athlete’s and coach’s knowledge for the promotion of optimal athlete health. This is also true for medical professionals, plus an understanding that athletes may omit positive usage of certain substances due to the preconceived condemnation from medical staff. The option of natural bodybuilding, consisting of excluding the use of AAS and other banned performance-enhancing drugs, could be a safer option, as no cases of AKI, CKD or ESKD were found to be published for this population.

## 5. Conclusions

Bodybuilders routinely engage in many nutritional and non-nutritional practices potentially harmful to the kidney (Figure 2). There is stronger evidence of damage caused by anabolic androgenic steroids and, particularly, high doses of vitamins A, D and E, than there is for high protein intake and creatine supplementation. Dehydrating practices including diuretic abuse and use of NSAIDs carry potential risks. Combinations of these practices are commonplace and may vary according to the training phase. Further research is required to identify the clinical and subclinical harm associated with individual practices and combinations, to enable the provision of appropriate advice.

## Figures and Tables

**Table 1:** Overview of Kidney disease and dietary and supplement history in 13 studies of Bodybuilders.

Study	Sex Age	Kidney Function at Presentation	Diagnosis	Protein	Creatine	Anabolic Steroids	Vitamins	Other
(Thorsteinsdottir, 2006)	Male 24	Screat 3.8 mg/dL	AIN (biopsy)	NS, Amino acid supplements	5 g × 3/week for 6 months	Denied	Yes	Diuretics-no NSAID-no
(Taner, 2011)	Male 18	Screat 202 umol/L Uprotein 0.28g/day	ATN (biopsy)	NS	L 20 g/day 5 days M 1 g/day 6 weeks	NS	NS	Diuretics-NS NSAID-NS
(Ardalan, 2012)	Male 32	Screat 4.3 mg/dL, Uprotein 0.85 g/day	AIN (biopsy)	NS	L 20 g/day 3 days M 1 g/day 3 weeks	NS	NS	Diuretics-NS NSAID-NS
(Almukhtar, 2015)	4 males, 20–26	Screat 230–336 µmol/L	All ATN (biopsy). 3 mild−moderate interstitial fibrosis	3.2–4.2 g/kg/day	L 15 g/day M 5 g plus 5 g pre- & 5 g post workout	Testosterone +/or nandrolone IM > 400 mg weekly	NS	Diuretics-NS NSAID-NS
(Hartung, 2001)	Male 27	Screat 1030 µmol/L, Uprotein 4532 mg/L	Nephrosclerosis, global glomerular sclerosis, CIN (biopsy)	2 g/kg/day	Creatine-210 g/day	Testosterone-750–1000 mg 6 weekly for 18 months	NS	Diuretics-no NSAID-no
(Herlitz., 2010)	10 males 28–49	Proteinuria- mean 10.1 g/d (1.3–26.3) Screat 3.0 mg/dL, (1.4–7.8 mg/dL)	FSGS (9) Glomerulomegaly (1) (All biopsy)	2.8–5.1 g/kg/day	Yes-dose NS	Various combinations of AAS	NS	Diuretics-NS NSAID-NS
(El-reshaid, 2018)	22 males 29 ± 7	Impaired kidney function or proteinuria and/or haematuria-NS	FSGS (8) Nephrosclerosis (4) AIN (2), CIN (3), Nephrocalcinosis (2), Membranous GN (1), Crescentic GN (1), Sclerosing GN (1) (All biopsy)	high-protein diet (20–30 g/kg/day)	NS	Testosterone 250 mg/day, Growth hormone (up to 100 mg/day)	NS	Diuretics-NS NSAID-NS
(Akl, 2019)	Male 26	Screat 12 mg/dL	FSGS (biopsy)	Yes-NS	Yes-NS	Yes-NS	NS	Diuretics-NS NSAID-NS
(Ali, 2020)	15 males 19–49	Screat 1.3–8.6 mg/dL	ATN (7), FSGS (2), AIN (1) Membranous GM (2) Nephrocalcinosis (2) Postinfectious GN (1) (All biopsy)	Yes-14/15 NS	Yes-13/15 NS	Yes-12/15 NS	Yes (13) Oral Vit D (11) Injected Vit D (2)	Diuretics-NS NSAID-NS
(Rocha, 2011)	Male 19	Scalcium 13.6 mg/dL Screat 2.64 mg/dL	Nephrocalcinosis	NS	NS	Denied	High dose Vit A, Vit D3, Vit E-over previous year	Diuretics-no NSAID-no
(Ronsoni 2017)	Male 24	Scalcium 13.6 mg/dL Screat 3.1 mg/dL	Nephrocalcinosis (Biopsy not done)	NS	NS	Growth hormone, nandrolone and other testosterone derivatives	High dose Vit A, Vit D3, Vit E-over previous year	Diuretics-NS NSAID-NS
(Libório, 2014)	Male 22	Scalcium 13.8 mg/dL Screat 8.6 mg/dL	Nephrocalcinosis, CIN (biopsy)	NS	NS	NS	High dose Vit D3 over previous 2 years	Diuretics-NS NSAID-NS
(Daher, 2017)	16 males 28 ± 9	Serum calcium 12 ± 2.2 mg/dL, Serum creatinine 3.9 ± 5.2 mg/dL	AKI (13) Nephrocacinosis (3) Nephrolithiasis (7) (biopsies not done) (Diagnoses not mutually exclusive)	NS	NS	Positive history of AAS (6). NS	High dose Vit A, Vit D3, Vit E-NS	Diuretics-NS NSAID-NS

NS = not specified, Screat = serum creatinine, Scalcium = serum calcium, AIN = acute interstitial nephritis, CIN = chronic interstitial nephritis, NSAID = non-steroidal anti-inflammatory agents, Uprotein = urinary protein, ATN = acute tubular necrosis, L = loading dose, M = maintenance dose, FSGS = focal segmental glomerular sclerosis, AAS = anabolic androgen steroids, Vit D = vitamin D, Vit D3 = vitamin D3, Vita A = vitamin A, Vit E = vitamin E. AKI = Acute kidney injury.

## Supplementary Materials

The following supporting information can be downloaded at: https://www.mdpi.com/article/10.3390/ijerph19074288/s1, Table S1: Overview of symptoms, renal measures, biopsy results, treatment, and subsequent renal diagnoses of bodybuilders presenting with AKI, CKD, and ESRD in 13 studies. Table S2: Overview of nutritional, pharmacological, and supplementary habits of bodybuilders presenting with AKI, CKD, and ESRD in 13 studies.
