# Supplementary material for: Nutritional and Non-Nutritional Strategies in Bodybuilding: Impact on Kidney Function
Source: Int J Environ Res Public Health. 2022 Apr 3;19(7):4288. doi: 10.3390/ijerph19074288 (PMC8998289; doi:10.3390/ijerph19074288)
Supplement: Supplementary file 1 [file ijerph-19-04288-s001.zip › Supp Material/TABLE S1.pdf]

Study	Participants	History of Kidney disease				Renal Biopsy results	Treatment	Diagnosis	AKI/ CKD/ ESRD
(Thorsteinsdottir, Grande, & Garovic, 2006)	24-year-old male	No	acute abdominal pain, polydipsia, polyuria	mg/24 hrs), hypertension (152/100 mm Hg), creatinine level (3.8 mg/dL), creatinine clearance (30 mL/min/24hrs), blood urea nitrogen (30 mg/dL)	Initial change: increased to 4.7 mg/dL Time to decrease: 5 days Final sCr: 2.5 mg/dL	12 normal glomeruli were identified where tubules were separated because of interstitial edema. Focal interstitial inflammatory infiltrates; lymphocytes + eosinophils identified.	all supplements discontinued	acute interstitial nephritis	
(Taner, Aysim, & Abdulkadir, 2011)	18-year-old male	No	nausea, vomiting and stomach-ache over the previous 2 days	Signs and symptoms at presentation	Initial measures	Serum Creatine levels (initial increase, final and time to decrease	all supplements discontinued and intravenous fluids	acute tubular necrosis	
(Ardalan, Samadifar, & Vahedi, 2012)	32-year-old male	No	2 weeks history of nausea and weakness	Hypertension (160/100 mmHg), sCr (4.3 mg/dL), serum urea (111 mg/dL), proteinuria and protein excretion clearance of 850 mg/24hrs	Initial change: 6.2 mg/dL Time to decrease: Not specified Final sCr: 1.8 mg/dL	normal glomeruli with mononuclear interstitial infiltrations and focal tubular disappearance plus few sloughed epithelial cells, and cellular debris in the tubular lumina	all supplements discontinued, Methylprednisolon pulse therapy (500 mg/day for 2 days) followed by 6 weeks of Prednisolon (60 mg/day)	Acute interstitial nephritis	
(Almukhtar, Abbas, Muhealdeen, & Hughson, 2015)	4 male BB (20-26years-old)	No	weakness and lethargy	Serum creatinine levels (229.84-335.92 µmol/L) or (2.6–3.8 mg/dL), eGFR (0.37-0.57 mL/s) or (22–34 mL/min) (Modification of Diet in Renal Disease calculation)	Initial change: Not specified Time to decrease: 4 weeks and 6 months Final sCr: At 4 wks- <123.76 µmol/L (1.4 mg/dL) At 6 months- 118.92, 88.40, 97.24 and 106.08 µmol/L (1.3, 1.0, 1.1 and 1.2 mg/dL)	Renal biopsies contained 12 to 16 glomeruli, and all biopsies revealed foci of flattened tubular epithelium with loss of nuclei and epithelial desquamation and blebbing. Hematoxylin staining amorphous deposits and occasional dense concretions were present within the injured tubules and adjacent interstitium. Patients 1 and 4 demonstrated interstitial fi-brosis and tubular atrophy (IF/TA) involving 30–40% of the cortex. Patient 2, IF/TA involved 10–15% of the cortex.	all supplements discontinued	acute tubular necrosis	

(Hartung, Gerth, Fünfstück, Gröne, & Stein, 2001)	27-year-old active BB	No	Periods of dizziness, fatigue, and vision loss over the previous 3months	Hypertension (190/110mmHg), sCr (1030 µmol/l), sCr clearance (10.2ml/min/1.73m^2), s-urea (31.6 mmol/l), protein excretion rate (4532 mg/l)	Not specified??	nephrosclerosis plus augmented global glomerulosclerosis and dispersed chronic tubulo-interstitial	haemodialysis treatment 3times/week	FSGS	
(Herlitz et al., 2010)	10 male BB (28 - 49 years)	No	6 out of 10 = systemic hypertension, 3 had a history of hypertension, all ranging 3months-5 years.<br>2 had sleep apnea	proteinuria (mean 10.1 g/d), mean sCr (3.0 mg/dl), sCr (1.4.7.8mg/dl), 24hrs-urine creatinine clearance (2.80-3.95 ml/min), 24-h protein (1.3 to 26.3 g/d)	Not specified??	Not specified??	renin–angiotensin system blockade treatments (n=7), corticosteroids (n=1)	FSGS (n=9)<br>Glomerulomegaly (n=1)	
(El-reshaid, El-reshaid, Al-bader, Ramadan, & Madda, 2018)	22 male BB (29 ± 7 years)	No	Not specified	impaired renal function or proteinuria and/or hematuria - Not specified	Not specified??	Not specified??	low-protein (0.4 g/kg/day/) diet, discontinue AAS, GH and (NSAIDs) use.<br>Treatments = angiotensin-converting enzyme or angiotensin-receptor antagonists	FSGS (n=8)<br>Nephroangiosclerosis (n=4)<br>Interstitial nephritis (n=7)<br>Also -<br>chronic interstitial nephritis (n=3),<br>acute interstitial nephritis (n=2),<br>nephrocalcinosis with chronic interstitial nephritis (n=2),<br>membranous glomerulopathy (n=1),<br>crescentic glomerulopathy (n=1),<br>sclerosing glomerulonephritis (n=1)	
(Akl & Aldabbagh, 2019)	26-year-old male BB	Not specified	referred due to blood donation unknowingly indicated low haemoglobin level	HB (8 g/dl),<br>sCr (12 mg/dl),<br>BUN (118 mg/dl)	Not specified	FSGS - severe renal cortical damage, glomerulosclerosis percentage was 100%	Intravenous fluids (partial improvement) and oral steroids (no response), initiated regular	FSGS	

							haemodialysis and kidney transplantation organised		
(Ali et al., 2020)	15 male BB (19-49years old)	Not specified	Not specified	elevated serum creatinine and/or proteinuria	Initial sCr – 1.3-8.6 mg/dL sCr after 1 year- 1.0-3.4 mg/dL (2 patients progressed to ESRD)	interstitial fibrosis and tubular atrophy – 0-70%	ATN- IV fluids and supplementation discontinuation<br>TIN - supplementation discontinuation<br>FSGS – Transplantation<br>PIGN and MGN – not specified<br>Nephrocalcinosis – transplantation and supplementation discontinuation	Acute tubular necrosis – 7/15<br>FSGS -2/15<br>MGN (membranous glomerulonephritis)- 2/15<br>Nephrocalcinosis - 2/15<br>TIN (tubulointerstitial nephritis) – 1/15<br>PIGN (post-infectious glomerulonephritis) – 1/15	
(Rocha, Santos, Avila, Neves, & Bahiense-Oliveira, 2011)	19-year-old BB	No	Anorexia, nausea, vomitting	Admission total serum calcium (13.6 mg/dL day 0),<br>peak total serum calcium (14.8 mg/dL day 3),<br>admission sCr (2.64mg/dL day 0),<br>peak sCr (2.88 mg/dL day 2),<br>outpatient sCr was 0.7 mg/dL three years ago,<br>25(OH) D level (150 ng/mL= toxic)	Initial change: Not specified<br>Time to decrease:<br>8 days<br>Final sCr: 1.09mg/dL	Not done	intense hydration and intravenous furosemide	Hypercalcaemia and AKI secondonary to vitamin D intoxication, At follow-up complete renal recovery	
(Ronsoni et al., 2017)	24-year-old BB	No	pain, nausea, fatigue, and vomiting for 2 months	sCr (3.1mg/dl),<br>25-OH-vitamin D (>150 ng/ml),<br>total calcium (13.6 mg/dl)	Initial change: Not specified<br>Time to decrease:<br>14 days<br>Final sCr:	Not done	intense hydration, furosemide and prednisone	hypercalcemia and ARF (acute renal failure) secondary to vitamin intoxication – check-up showed normal laboratory results plus increased 25-OH- vitamin D of 107 ng/mL	

(Libório, Nasserala, Gondim, & Daher, 2014)	22-year-old BB	No	nausea, vomiting, and upper abdominal pain in the past 5 days	lipase levels (218U/l), haemoglobin levels (7.8g/dl), sCr (8.6mg/dl), serum calcium (13.8mg/dl)	4 days post haemodialysis and prednisone = reduction in serum calcium, Two weeks after acute pancreatitis recovery = normalisation of serum calcium while serum creatinine of 1.8mg/dl remained	moderate interstitial fibrosis and tubular atrophy surrounding numerous interstitial calcium deposits	intense hydration and intravenous furosemide - no reduction in serum calcium levels and as renal failure and hypercalcaemia persisted, haemodialysis and prednisone	CKD- Nephrocalcinosis	
(Daher et al., 2017)	16 males (28.3 ± 8.9 years)	No	headaches, nausea, vomiting and abdominal pain	sCr (3.9±5.2mg/dl), serum calcium (12 ±2.2mg/dl), Peak serum calcium (mean; 13 ± 2.2 mg/dL, ranging 10.2 to 16.8 mg/dL), serum vitamin D (135±75 ng/ml),	Peak sCr (mean; 4.3 ± 5.2 mg/dL, ranging 0.8 to 8.6 mg/dL),	Not specified	intense hydration, steroids, and loop diuretics, 2 cases of severe AKI – dialysis, 2 cases required haemodialysis Mean length of hospitalization - 15 ± 13 days (1–52 days)	3 cases of nephrocalcinosis, 7 revealed nephrolithiasis, resulted in 13/16 cases deemed as AKI secondary to hypercalcaemia, 1 case progressed to CKD	
