# Supplementary material for: Nutritional and Non-Nutritional Strategies in Bodybuilding: Impact on Kidney Function
Source: Int J Environ Res Public Health. 2022 Apr 3;19(7):4288. doi: 10.3390/ijerph19074288 (PMC8998289; doi:10.3390/ijerph19074288)
Supplement: Supplementary file 1 [file ijerph-19-04288-s001.zip › Supp Material/TABLE S2.pdf]

Study	Protein	Creatine	Anabolic steroids	Vitamins	NSAIDS	Diuretics/laxatives	Other
(Thorsteinsdottir, Grande, & Garovic, 2006)	Not specified<br>Amino acid supplements	5g- x3/week for 6 months	Denied	Yes	Denied	Denied	multiple herbs & non-herbal supplements
(Taner, Aysim, & Abdulkadir, 2011)	Not specified	Loading = 20g/day for 5 days<br>Maintenance = 1g/day for 6 weeks	Not specified	Not specified	Not specified	Not specified	Not specified
(Ardalan, Samadifar, & Vahedi, 2012)	Not specified	Loading = 20g/day for 3 days<br>Maintenance = 1g/day for 3 weeks	Not specified	Not specified	Not specified	Not specified	Not specified
(Almukhtar, Abbas, Muhealdeen, & Hughson, 2015)	3.2–4.2 g/kg/day of protein: 78–104 g of whey powder + 2-3L/day milk	15g/day – 5g maintenance, 5g pre and 5g post workout	Testosterone proprionate +/or nandrolone deconate IM >400mg weekly	Not specified	Not specified	Not specified	Not specified
(Hartung, Gerth, Fünfstück, Gröne, & Stein, 2001)	High protein diet – 2g/kg/day	Creatine -210g/day	testosterone -750–1000 mg/week<br>6 week intervals over 18 months	Not specified	Denied	Denied	Not specified
(Herlitz et al., 2010)	Yes – 8/10<br>550 g/d protein<br>300-400g/day<br>500g/day<br>300g/day<br><br>Mean body weight – 107Kg<br>Mean protein-2.8-5.1g/Kg/day	Yes – dose not specified	Combinations of - testosterone, prohormone, durabolin, stanazolol, sustanon, primobolan, equipoise, and winstrol<br>Specific quantities not specified	Not specified	Not specified	Not specified	Not specified
(El-reshaid, El-reshaid, Al-bader, Ramadan, & Madda, 2018)	high-protein diet (20–30 g/kg/day)	Not specified	Testosterone ~250mg/day, Growth hormone (up to ~100mg/day)	Not specified	Not specified	Not specified	Not specified
(Akl & Aldabbagh, 2019)	high protein diet – not specified	Yes – not specified	Positive history - Not specified	Not specified	Not specified	Not specified	Energy drinks for rehydration

(Ali et al., 2020)	Yes – 14/15 not specified	Yes – 13/15 not specified	Yes – 12/15 not specified	Yes – 13/15<br>Oral intake Vit D – 11/15<br>Injection Vit D – 2/15	Not specified	Not specified	Not specified
(Rocha, Santos, Avila, Neves, & Bahiense-Oliveira, 2011)	Not specified	Not specified	Denied	300mL over the previous year<br>Per 100ml:<br>Vit A- 20,000,000IU<br>Vit D3- 5,000,000IU<br>Vit E- 6,800IU	Denied	Denied	several over- the-counter food supplements – not speficied
(Ronsoni et al., 2017)	Not specified	Not specified	Postive history of growth hormone, nandrolone and other testosterone derivatives	150ml over the last 4 months<br>Vit A- 20,000,000IU<br>Vit D3- 5,000,000IU<br>Vit E- 6,800IU	Not specified	Not specified	Not specified
(Libório, Nasserala, Gondim, & Daher, 2014)	Not specified	Not specified	Not specified	1 x 50mL /month over 2years cholecalciferol- 50,000IU of vitamin D per mL	Not specified	Not specified	Not specified
(De Francesco Daher et al., 2017)	Not specified	Not specified	Positive history of AAS (n=6)<br>Quantity- not specified	positive history<br>Vit A- 20,000,000IU<br>Vit D- 35,000,000IU<br>Vit E- 6,000IU<br>Per 100ml<br>Quantity not specified	Not specified	Not specified	Not specified
